# Epigenome-wide association study reveals decreased average methylation levels years before breast cancer diagnosis

**DOI:** 10.1186/s13148-015-0104-2

**Published:** 2015-08-04

**Authors:** Karin van Veldhoven, Silvia Polidoro, Laura Baglietto, Gianluca Severi, Carlotta Sacerdote, Salvatore Panico, Amalia Mattiello, Domenico Palli, Giovanna Masala, Vittorio Krogh, Claudia Agnoli, Rosario Tumino, Graziella Frasca, Kirsty Flower, Ed Curry, Nicholas Orr, Katarzyna Tomczyk, Michael E. Jones, Alan Ashworth, Anthony Swerdlow, Marc Chadeau-Hyam, Eiliv Lund, Montserrat Garcia-Closas, Torkjel M. Sandanger, James M. Flanagan, Paolo Vineis

**Affiliations:** MRC-PHE Centre for Environment and Health, Imperial College London, London, W2 1PG UK; HuGeF Foundation, 52, Via Nizza, Torino, 10126 Italy; Departimento di Medicina Clinica e Chirurgia, Federico II University, Naples, Italy; Molecular and Nutritional Epidemiology Unit, Cancer Research and Prevention Institute—ISPO, Florence, Italy; Epidemiology and Prevention Unit Fondazione IRCCS Istituto Nazionale dei Tumori, Milan, Italy; Cancer Registry ASP, Ragusa, Italy; Epigenetics Unit, Division of Cancer, Department of Surgery and Cancer, Faculty of Medicine, Imperial College London, 4th Floor IRDB, Hammersmith Campus, Du Cane Road, London, W12 0NN UK; Breakthrough Breast Cancer Research Centre, The Institute of Cancer Research, London, UK; Division of Breast Cancer Research, The Institute of Cancer Research, London, UK; Division of Genetics and Epidemiology, The Institute of Cancer Research, London, UK; Department of Community Medicine, UiT—the Arctic University of Norway, Tromsø, Norway

**Keywords:** EWAS, Methylation, Risk, Biomarker, Breast cancer, Peripheral blood

## Abstract

**Background:**

Interest in the potential of DNA methylation in peripheral blood as a biomarker of cancer risk is increasing. We aimed to assess whether epigenome-wide DNA methylation measured in peripheral blood samples obtained before onset of the disease is associated with increased risk of breast cancer. We report on three independent prospective nested case-control studies from the European Prospective Investigation into Cancer and Nutrition (EPIC-Italy; *n* = 162 matched case-control pairs), the Norwegian Women and Cancer study (NOWAC; *n* = 168 matched pairs), and the Breakthrough Generations Study (BGS; *n* = 548 matched pairs). We used the Illumina 450k array to measure methylation in the EPIC and NOWAC cohorts. Whole-genome bisulphite sequencing (WGBS) was performed on the BGS cohort using pooled DNA samples, combined to reach 50× coverage across ~16 million CpG sites in the genome including 450k array CpG sites. Mean *β* values over all probes were calculated as a measurement for epigenome-wide methylation.

**Results:**

In EPIC, we found that high epigenome-wide methylation was associated with lower risk of breast cancer (odds ratio (OR) per 1 SD = 0.61, 95 % confidence interval (CI) 0.47–0.80; −0.2 % average difference in epigenome-wide methylation for cases and controls). Specifically, this was observed in gene bodies (OR = 0.51, 95 % CI 0.38–0.69) but not in gene promoters (OR = 0.92, 95 % CI 0.64–1.32). The association was not replicated in NOWAC (OR = 1.03 95 % CI 0.81–1.30). The reasons for heterogeneity across studies are unclear. However, data from the BGS cohort was consistent with epigenome-wide hypomethylation in breast cancer cases across the overlapping 450k probe sites (difference in average epigenome-wide methylation in case and control DNA pools = −0.2 %).

**Conclusions:**

We conclude that epigenome-wide hypomethylation of DNA from pre-diagnostic blood samples may be predictive of breast cancer risk and may thus be useful as a clinical biomarker.

**Electronic supplementary material:**

The online version of this article (doi:10.1186/s13148-015-0104-2) contains supplementary material, which is available to authorized users.

## Background

Differences in DNA methylation observed in human tumour tissue compared to normal tissue were reported 30 years ago [1]. Early reports showed hypomethylation of oncogenes in several carcinomas versus healthy tissues [2, 3]. Numerous studies since have established that hypermethylation, mainly of CpG islands (CGIs) on promoters of tumour suppressor genes [4, 5], and global (or genome-wide) hypomethylation in tumours relative to non-tumorous tissues occur in a wide variety of cancers [6, 7].

Despite the fact that most studies have measured global methylation in repetitive elements, other studies suggest that hypomethylation in cancer is not just limited to repeats but also occurs in gene regions [8–10]. In tumour DNA, Irizarry et al. found hypomethylation of CpG shores, but not of CpG islands, and Hansen et al. reported hypomethylated blocks across the epigenome [11, 12]. It was not the presence of repetitive sequences but rather of these hypomethylated blocks across unique sequences, which caused most of the overall hypomethylation in tumours [11, 12]. For this reason, we hypothesised that it would be possible to use the Illumina Infinium HumanMethylation450 (HM450) BeadChip array to assess genome-wide methylation levels. This array measures DNA methylation at approximately 485,000 CpG sites distributed across the entire genome, including CpGs on islands, shores, and shelves, as well as gene promoters and bodies, intergenic regions, and other areas [13]. This covers ~1.5 % of the 28 million CpG sites known in the genome.

In the last few years, there has been increasing interest in using blood samples to measure DNA methylation in cancer cases and controls [14, 15]. The most robust candidate gene studies have used pre-diagnostic blood samples to report associations between breast cancer risk and methylation of *ATM* and *BRCA1* genes [16–18]. However, most previously conducted studies—including genome-wide studies—have been retrospective, cross-sectional studies. A recent review and meta-analysis concluded that there could be great potential for DNA methylation in peripheral white blood cells (WBCs) as a biomarker for cancer risk when total 5-methylcytosine levels were measured; however, methylation measured by surrogate assays for repetitive elements was not associated with cancer risk, and factors such as study design and data analysis methods were often suboptimal [19, 20]. In addition, two other reviews highlighted challenges such as sample selection and population choice when planning epigenome-wide association studies (EWAS) [21, 22].

In the current study, we describe the results of nested case-control studies from three prospective cohorts in which we measured genome-wide methylation in peripheral WBCs of subjects who later developed breast cancer compared to subjects who remained cancer free during follow-up. We also compare our results with a recent report from the Melbourne Cancer Cohort Study (MCCS) that has used the same Illumina 450k methodology as our study and reported a significant association between epigenome-wide methylation and breast cancer risk (odds ratio (OR) per 1 SD = 0.69 (0.50–0.95, *p* = 0.02) [23]. We estimated genome-wide methylation from the 450k methylation array and from overlapping CpG sites in whole-genome bisulphite sequencing, positing that genome-wide hypomethylation may be present before diagnosis and could be useful as a biomarker for early detection or risk of breast cancer.

## Results

### Epigenome-wide hypomethylation is associated with risk of breast cancer

**Fig. 1:**
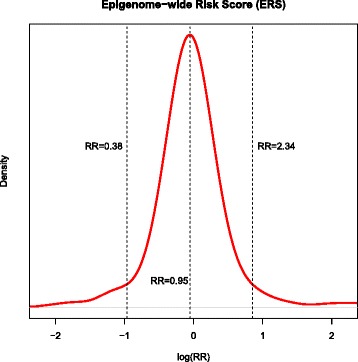
Log relative risk distribution of individuals in EPIC using the effect estimate of the logistic model of splined global methylation as it relates to case-control status. The log (RR) is presented on the *x-axis*, with density on the *y-axis* and with the median and 95 % range marked with the *dotted line*

Using EPIC-Italy, the first data set we investigated, the mean *β* value (all probes) between matched breast cancer cases (53.00 %) and controls (53.18 %) was 0.18 % lower in cases (paired Wilcoxon test *p* = 1.82e−05). The median methylation values in cases (65.15 %) and controls (65.67 %) were also lower in cases (0.5 %, *p* = 1.33e−06). Conditional logistic regression analysis using categorical methylation in quartiles is reported in Table 1 and shows a marked decrease of breast cancer risk with increasing mean *β* values. The analyses of the per-quartile median methylation provided an estimate of the OR for 1 SD increase in methylation (OR = 0.61, 95 % confidence interval (CI) 0.47–0.80, *p* = 0.0004) (Table 1). Using the more conservative robust logistic regression, we confirmed the observation of lower methylation in cases compared with controls (OR per 1 SD = 0.71, 95 % CI 0.61–0.84, *p* = 0.00003). Adjusting for white blood cell composition or removal of probes affected by cell type did not materially change the results (Additional file 1: Table S1). Linear regression models of epigenome-wide methylation versus well-established breast cancer risk factors did not show any association (Additional file 1: Table S2), supporting the notion that epigenome-wide methylation is independent of these factors. We have performed the receiver operating curve (ROC) analysis to assess the classification performance of average DNA methylation levels to predict breast cancer case status, which showed an AUC of 62 % (95 % CI 56–68 %). From the B-spline regression model of continuous levels of genome-wide methylation in EPIC, we estimated the distribution of individual risk in this population (95 % range RR 0.38–2.34) (Fig. 1). In contrast to EPIC, overall genomic hypomethylation was not associated with increased risk of breast cancer in the NOWAC cohort (OR per 1 SD = 1.03 (95 % CI 0.82–1.30), *p* = 0.81) using all available data (Table 1) or only the probes that overlap both datasets (Additional file 1: Table S3). Similarly, the mean methylation in cases (54.02 %) and controls (54.02 %) and median methylation levels (68.67 % vs 68.70 %) were not significantly different in NOWAC (*p* = 0.79).

**Table 1:** Association between average methylation and breast cancer risk in EPIC and NOWAC

		Cases (*n*)	Controls (*n*)	OR	(95 % CI)	*p* value
EPIC						
By quartile	Q1 [0.529–0.546]	75	41	1.00		
	Q2 [0.546–0.549]	31	40	0.46	(0.25–0.84)	0.01
	Q3 [0.549–0.551]	30	40	0.40	(0.21–0.76)	0.005
	Q4 [0.551–0.560]	26	41	0.34	(0.18–0.66)	0.001
	Per 1 SD	162	162	0.61	(0.46–0.80)	0.0003
Time to diagnosis	<3.8	81	81	0.66	(0.46–0.94)	0.02
(years)	>3.8	81	81	0.54	(0.35–0.83)	0.005
				*p* het = 0.483		
ER status	Negative	18	18	0.49	(0.20–1.24)	0.13
	Positive	56	56	0.59	(0.36–0.96)	0.03
				*p* het *=* 0.725		
NOWAC						
By quartile	Q1 [0.527–0.538]	45	42	1.00		
	Q2 [0.538–0.540]	32	42	0.74	(0.41–1.34)	0.32
	Q3 [0.540–0.543]	46	42	1.04	(0.59–1.85)	0.88
	Q4 [0.543–0.551]	45	42	0.99	(0.56–1.76)	0.98
	Per 1 SD	168	168	1.03	(0.82–1.30)	0.81
Time to diagnosis	<2.1	84	84	0.92	(0.66–1.29)	0.62
(years)	>2.1	84	84	1.15	(0.83–1.60)	0.41
				*p* het = 0.351		
ER status	Negative	28	28	0.80	(0.48–1.32)	0.38
	Positive	130	130	1.10	(0.84–1.44)	0.50
				*p* het *= 0.276*

**Fig. 2:**
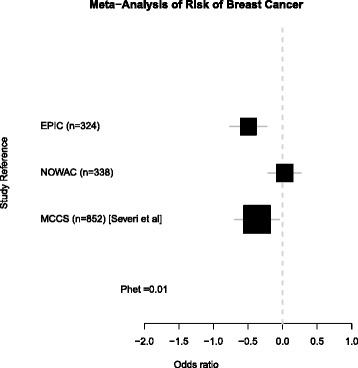
Forest plot meta-analysis of three independent breast cancer case-control studies. The effect estimates are derived from the “per 1 SD odds ratio” and presented as a log of odds ratio. The *p* value for heterogeneity is *p* = 0.01 indicating significant heterogeneity in the populations. The number of subjects (cases and controls) in each study is reported. Data from Severi et al. [23] have been reported elsewhere

In Table 2, we combined the mean and standard deviations from these two studies with a previously published report from the MCCS study [23]. A meta-analysis of all three breast cancer 450k studies (EPIC, NOWAC, and MCCS) showed significant heterogeneity between studies (*p* het = 0.01) (Fig. 2). We also used whole-genome bisulphite sequencing (WGBS) data from a fourth independent cohort, the BGS, to validate the main findings from the Illumina 450k analysis. Similar to the 450k array in EPIC, we observed a 0.2 % mean hypomethylation in breast cancer cases compared with controls across the same sites in the 450k array (Table 2).

**Table 2:** Average methylation and breast cancer risk in four studies

Study	Method	Cases			Controls			Diff
		Mean (%)	SD (%)	IQR	Mean (%)	SD (%)	IQR	(%)
EPIC	450k	53.00	0.39	[52.68–53.27]	53.18	0.35	[52.97–53.40]	−0.18
NOWAC	450k	54.02	0.45	[53.73–54.32]	54.02	0.41	[53.77–54.29]	0.00
MCCS^a^	450k	51.86	1.00	nd	51.95	1.01	nd	−0.09
BGS^b^	WGBS	48.12	–	–	48.30	–	–	−0.18

*nd* not done (not reported)

^a^[23]

^b^Flanagan and Garcia-Closas, unpublished data

**Fig. 3:**
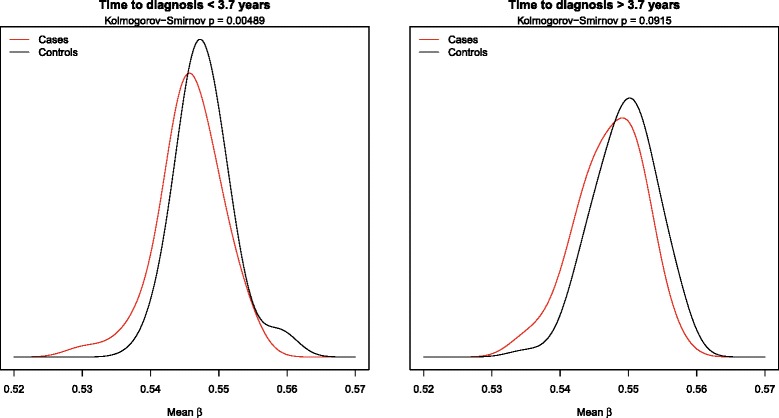
Kernel density estimate for samples collected less than 3.7 years before diagnosis and more than 3.7 years before diagnosis in EPIC. The *p* values refer to the significance level of the Kolmogorov-Smirnov test of equality in distribution between cases and controls.

We then conducted a more detailed analysis of the EPIC dataset in which we observed the association with breast cancer risk. We found that time from blood draw to diagnosis (below or above the median) in EPIC did not seem to influence the estimate of association between genome-wide methylation and breast cancer risk (Table 1, Additional file 2: Figure S1, Fig. 3) (test for heterogeneity by time to diagnosis, *p* = 0.45). Furthermore, we have performed the analysis separately for subjects with a time to diagnosis in EPIC of <1 year (*n* = 20, OR = 0.23 (0.06–0.86), *p* = 0.03) and subjects >1 year (*n* = 142, OR = 0.56 (0.40–0.80), *p* = 0.001) with both showing similar results.

To investigate which probe types contribute most to the difference in methylation between cases and controls in the EPIC population, we stratified the association between epigenome-wide methylation and breast cancer risk for different groups of probes based on location or function (Table 3). Excluding SNP probes and cross-hybridising probes did not change the results. The mean methylation level of probes located on gene promoters was not associated with breast cancer risk (*p* = 0.66). However, probes on gene bodies or at the 3′UTR were both significantly hypomethylated in cases compared with controls (*p* < 2 × 10^−5^). We also observed this difference in the WGBS data which showed hypomethylation in cases compared with controls in gene body CpG sites (66.1 % vs 66.5 %, −0.4 %) in contrast to CpG islands that were not different (18.7 % vs 18.7 %, 0.0%). Corresponding results for NOWAC are reported in Additional file 1: Table S4 and Table S5.

**Table 3:** Association between global methylation and breast cancer risk by CpG genomic feature per 1 SD in EPIC

		# CpG loci	OR	(95 % CI)	*p* value
All	Including all probes	408,749	0.61	(0.47–0.80)	0.0004
	Excluding SNP probes	360,342	0.62	(0.47–0.81)	0.0004
CpG island	Island	124,962	0.76	(0.57–0.99)	0.04
	Shores	98,890	0.72	(0.55–0.93)	0.01
	Shelves	38,755	0.50	(0.37–0.68)	8.93 × 10^−6^
	None	146,142	0.50	(0.38–0.68)	7.44 × 10^−6^
Gene region feature category	TSS1500	59,494	0.70	(0.53–0.92)	0.01
	TSS200	43,506	0.92	(0.69–1.24)	0.60
	5′UTR	36,778	0.72	(0.54–0.95)	0.02
	1st exon	19,024	0.85	(0.65–1.12)	0.25
	Promoter	82,006	0.92	(0.64–1.32)	0.66
	Gene body	138,499	0.51	(0.38–0.69)	1.58 × 10^−5^
	UTR3	15,065	0.40	(0.29–0.57)	2.90 × 10^−7^
	Intergenic	96,383	0.57	(0.43–0.75)	6.86 × 10^−5^

We performed a principal component analysis on the samples (i.e., using the transpose matrix of the normalised *M*-value methylation profiles). In EPIC, we found that the first component was associated mostly with age (*p* = 0.00012) and menopausal status (*p* = 0.002), the second component (PC2) associated with case-control status (*p* = 0.0005) and dietary folate levels (*p* = 0.0006), while the third component (PC3) associated mostly with BMI (*p* = 0.01) and weight (*p* = 0.01) (Table 4). In NOWAC, we observed an association with menopausal status and components of methylation variability (PC1 *p* = 0.08, PC2 *p* = 0.02) (Additional file 1: Table S6).

**Table 4:** Association between principal components and subject variables in EPIC

	First PC	Second PC	Third PC
% of variance explained	0.064	0.034	0.022
Minimum *p* value chips	0.327	0.124	0.516
Covariates			
Case/control status	0.02	*0.0005*	0.23
Age	*0.00012*	0.63	0.16
Weight	0.84	0.10	0.01
Height	0.73	0.59	0.60
BMI (continuous)	0.74	0.14	0.01
BMI (categorical)			
Underweight	0.50	0.11	0.44
Overweight	0.86	0.11	0.34
Obese	0.96	0.20	0.01
Physical activity (cat)			
Moderately inactive	0.05	0.17	0.57
Moderately active	0.03	0.01	0.38
Active	0.43	0.73	0.76
Red meat	0.02	0.08	0.90
Alcohol	0.23	0.27	0.40
Folate	0.91	*0.0006*	0.72
Smoking			
Former	0.77	0.72	0.72
Current	0.15	0.20	0.93
Age at menarche (cat)			
12–14	0.53	0.03	0.16
≥15	0.20	0.41	0.88
Age at menopause	0.45	0.73	0.25
Menopausal state	*0.002*	0.25	0.78
Ever pill	0.21	0.62	0.18
Ever HRT	0.34	0.09	0.60
ER status	0.42	0.14	0.39
PR status	0.76	0.44	0.08

To demonstrate that there was no batch effect for the chip, we report the smallest *p* value for the association between the PCs and all chips.

Italics = *p*-values <0.01

### Comparison of study-specific probe signatures associated with breast cancer risk

In the EPIC cohort using conditional logistic regression, we identified 26 probes significantly associated with breast cancer risk (*p* < 1.2 × 10^−7^) (Additional file 1: Table S7), and in the NOWAC study, we identified 0 significant probes (*p* < 1.2 × 10^−7^) and could not replicate the 26 probes identified in EPIC. Similarly to a previous study [24], we found that the majority of probes were hypomethylated in cases compared with controls in the EPIC cohort, consistent with the overall epigenome-wide hypomethylation.

## Discussion

In this study, we report genome-wide hypomethylation among breast cancer cases compared with matched controls in three out of four cohorts using the Illumina 450k array and WGBS. Specifically, in EPIC-Italy, hypomethylation was observed in gene body probes but not in gene promoters. This association was not associated with time to diagnosis indicating that it is unlikely to be attributable to an early process of carcinogenesis. We have further evaluated these findings using WGBS of pooled DNA samples from cases and controls. Results were consistent with overall genome-wide hypomethylation in cases compared to controls, specifically in gene body sequences compared with CpG islands. Principal component analysis in the EPIC cohort highlighted other factors that may impact on genome-wide methylation, such as age, menopausal status, and folate levels.

The significant heterogeneity between the three Illumina 450k studies was primarily driven by results from the Norwegian population (NOWAC) that differed from those in the Italian (EPIC) or Australian (MCCS) populations. This could be explained by differences in the distribution of environmental, lifestyle, or other subject characteristics. We observed differences in the distribution of several breast cancer risk factors between EPIC and NOWAC, which might explain the heterogeneity of results, including mean age, weight, height, smoking status, and menopausal status (Additional file 1: Table S8). There is also a significant difference in follow-up time in EPIC (mean 8.9 years (range 0.04–15.7 years)) compared to NOWAC (mean 4.8 years (range 3.1–6.6 years)) (Additional file 1: Table S8). However, further studies will be needed to confirm the association between epigenome-wide methylation and breast cancer risk and the possible modification by menopausal status which was associated with principle components of methylation variation in both NOWAC and EPIC.

Previous studies assessing peripheral blood DNA methylation and breast cancer risk have produced inconsistent results. Most studies assessing “global methylation” used a retrospective or cross-sectional design and did not measure sequence-specific genome-wide methylation, but rather methylation in various repetitive elements (such as LINE-1, ALU, and Sat2) as a surrogate measure, using different types of assays and methods, making it difficult to compare results across studies [19, 20]. One of the few large prospective breast cancer studies that assessed genome-wide levels of LINE-1 DNA methylation in three independent cohort studies (each consisting of >200 cases and >200 controls) using pre-diagnostic blood samples concluded that there was no difference between cases and controls in LINE1 methylation, even after adjustment for confounding [16]. In contrast to this, a recent report from the prospective Sister Study (*n* = 294 cases) shows hypomethylation in LINE1 associated with breast cancer risk [25]. These conflicting results suggest that new standardised methods are required to interpret and analyse epigenome-wide methylation using repetitive element assays [26].

Overall, the mean genome-wide methylation level is ~0.2 % lower in cases compared to controls, which may be interpreted as representing a larger difference in a smaller proportion of probes. Several studies have previously reported genome-wide signatures of breast cancer using the 450k array [27] or the predecessor 27k array [24, 28]. Using the 27k array, one study reported 250 CpG sites to be differentially methylated between 289 cases and 612 controls in pre-diagnostic blood samples, of which the majority (75%) were hypomethylated in cases compared with controls [24]. Another report identified a 92-probe signature (FDR *q* < 0.05) with a larger signature of *n* = 1850 probes (raw *p* < 0.037) also reported in the study [28]. Using our data from the 450k array, we have attempted to replicate independently the specific probes reported in these two 27k studies (with the majority of 27k probes also present on the 450k array) but have failed to find any overlap between the two 27k studies [24, 28] and the two 450k studies (EPIC and NOWAC), with the direction of changes not significantly different to chance for these probes. These differences may be attributable to differences in the subject populations and tumour pathologies but most likely due to low power in each of these studies. These data indicate that the study-specific signatures reported here and in other reports do not yet converge on a robust and validated set of individual probes further supporting the need to increase the study sizes to identify robustly individual CpG sites associated with breast cancer risk before proceeding with extensive validation of these top hits.

While the majority of investigators have predominantly used whole blood DNA for epigenetic epidemiology studies, it is well known that the epigenetic state for various subsets of CG sites in the genome are dependent on blood cell type, age, and various exposures [19]. While various methods can be used to account for each of these possibilities, such as excluding the probes affected or adjusting for the confounders, these are not always perfect. Our results suggest that the association with risk is unlikely to be explained by a different white blood cell composition among cases and controls as there was no change in the results with or without accounting for blood cell type. However, these analyses do not adjust for immune cell activation and clonal expansion which might also contribute to epigenetic variation in white blood cell DNA samples as reported recently [29]. The most appropriate study design to address these limitations would be to collect blood samples and sort into different cell types prior to storage in a prospectively collected cohort with many years of follow-up to accumulate incident cancer cases.

We observed hypomethylation for CpGs located on shores and shelves of CpG islands and in gene bodies but not in promoters, supporting the lack of variability in CpG island promoters [12]. Like many previous studies, we also observed hypomethylation of probes that map to all categories of repetitive elements (data not shown). However, our observation of increasing hypomethylation across the whole genome with increasing breast cancer risk, measured both continuously and categorically, supports the hypothesis that hypomethylation is not restricted to repetitive elements but includes all areas of the genome [11, 30]. One hypothesis for a mechanism driving this hypomethylation is a general deficiency in methylation enzymes or substrates due to the complex interaction between folate, alcohol use, and one-carbon metabolism genes in relation to breast cancer risk [31] and methylation [32]. While we show an association between genome-wide methylation and folate levels in EPIC (Additional file 1: Table S2, *p* = 0.04), further validation of this finding is needed to support this hypothesis.

## Conclusions

In conclusion, the results of this study indicate that genome-wide hypomethylation, measured in pre-diagnostic blood samples using the Illumina HM450 array or by WGBS, could predict breast cancer risk. However, additional studies with larger sample sizes, WBC counts, as well as additional breast cancer risk factor information including genetic factors are needed to evaluate its potential value as an independent risk biomarker.

## Availability of supporting data

The EPIC data set supporting the results of this article is available in the Gene Expression Omnibus (GEO) repository, accession GSE51057.

## Methods

### Participants

For this study, we have used three independent cohorts in which we have selected incident breast cancer cases compared to matched cancer-free controls in a nested case-control study design. These were the Italian cohort of the European Prospective Investigation into Cancer and Nutrition (EPIC) study (*n* = 166 pairs) [33], the Norwegian Women and Cancer (NOWAC) study (*n* = 192 pairs) [34], and the Breakthrough Generations Study (BGS) (*n* = 548 pairs) [35]. All study participants signed informed consent forms, and each cohort was approved by the national ethical review boards.

#### EPIC

Participants for this nested case-control study were selected from the Italian cohort of the European Prospective Investigation into Cancer and Nutrition (EPIC) study. This sub-cohort consists of 46,857 volunteers (including 32,157 women), recruited from 5 different centres within Italy (Varese, Turin, Florence, Naples, and Ragusa) [33]. Incident cases were identified through cancer registries with <2 % losses to follow-up. We identified 166 incident female breast cancer cases, for each of which we collected 166 healthy female controls (matched on date of birth (±5 years), month of recruitment and study centre). Average follow-up (cases and controls combined) was 106.8 months (range: 0.53–188.8 months) and average time to diagnosis was 63.4 months (range 0.53–187.8). Main features of the resulting study population are summarised in Additional file 1: Table S1. For all study participants, detailed baseline information about lifestyle habits and personal and family history was collected through questionnaires, along with blood samples and anthropometric measurements at enrolment between 1993 and 1998. All participants signed an informed consent form, and the ethical review boards of the International Agency for Research on Cancer (IARC) and of local participating centres approved the study protocol.

#### NOWAC

Participants for this nested case-control study were selected from the Norwegian Women and Cancer (NOWAC) study [34]. This study recruited from 1991 to 2006 and collected questionnaire information from 170,000 women with repeated collection of information after 4–6 years (2 or 3 times) and a biobank of more than 50,000 blood samples from participants in 2003–2006. Incident breast cancer cases were identified through the Norwegian Cancer Registry. We selected 192 incident female breast cancer cases, matched to 192 healthy female controls (matched on birth year and month of recruitment). Average time to diagnosis was 25.2 months (range 0–60). Main features of the resulting study population (*n* = 336) are summarised in Additional file 1: Table S2, and differences between the NOWAC and EPIC cohorts are described in Additional file 1: Table S3. All participants signed an informed consent form, and the NOWAC study was approved by the Regional Committee for Medical and Health Research Ethics in North Norway.

#### BGS

The Breakthrough Generations Study (BGS) is a large general population cohort consisting of ~110,000 women enrolled in the UK from 2003 to 2011 [3]. For the methylation analyses, we have selected DNA samples from a case-control study nested in the BGS cohort. The inclusion criteria are as follows. We initially selected all confirmed incident cases and matched controls at the time of selection that met the following criteria: white ethnicity, subjects not related to another previously selected enrolled participant (first family member recruited), provided a blood sample received at the processing laboratory in post <2 days after collection, sample not clotted, and with available DNA extracted from buffy coats at concentration >40 ng/μL. Controls were individually matched to cases on age, ethnicity, and date of recruitment. This resulted in a total of 916 case-control pairs from whom we selected a random sample of 548 case-control pairs to make four DNA pools of cases and four DNA pools for their matched controls. We stratified the DNA samples into four pathology sub-groups (123 cases with in situ tumours, 66 cases with invasive estrogen receptor (ER)-negative tumours, 179 cases with invasive ER-positive tumours with early onset (age at diagnosis <50 years), and 189 cases with invasive ER-positive tumours with late onset (age at diagnosis >50 years)). Although all cases had a date of diagnosis after blood collection at the time of selection, subsequent record updates identified one case in the in situ pool diagnosed 2 years prior to blood collection, two cases in the in situ and ER-negative pools diagnosed 22 days prior to blood collection, and one case in the ER-positive late onset cancers with a previous diagnosis of in situ cancer 22 years prior to the diagnosis of the invasive cancer. Due to the pooling nature of this experiment, these few subjects cannot be excluded from analyses; however, they are unlikely to change the overall results. Each pool included 200 ng of peripheral blood DNA from each of the subjects to make a pooled DNA sample that was subsequently processed for library preparation and sequencing. Main features of the resulting study population (*n* = 548 cases and 548 matched controls) are summarised in Additional file 1: Table S4. All BGS participants signed an informed consent form, and the study was approved by the South East Research Ethics Committee (NREC 03/1/014).

### DNA methylation measurement, data pre-processing, and quality control for 450k arrays

DNA extractions and methylation array processing were conducted in the same laboratory (HuGeF, Torino, Italy) for both the EPIC and NOWAC studies. DNA was extracted from buffy coats or blood cell fractions using the QIAsymphony DNA Midi Kit (Qiagen, Crawley, UK). Five hundred nanograms of DNA were bisulphite-converted with the EZ-96 DNA Methylation-Gold™ Kit (Zymo Research, Orange, CA, USA) according to the manufacturer’s protocol. Next, the Illumina Infinium HumanMethylation450 BeadChip was hybridised as per the manufacturer’s protocol. This array measures DNA methylation at 485,512 cytosine positions across the human genome, of which 482,421 CpG sites and 3091 non-CpG sites; hereafter, the term CpG will be used to refer to all of these, unless otherwise specified. BeadChips were washed and scanned using the Illumina HiScan SQ scanner, and intensities were extracted from the images using GenomeStudio (v.2011.1) and its Methylation module (1.9.0). Bisulphite conversion efficiency was assessed using control probes present on the chip, failing samples outside 3 SD of the sample distribution; all samples passed this initial quality control step. Additional pre-processing included background subtraction and colour correction to account for the dye bias seen in Infinium II probes. This was done by equalising the intensities in the green and red channels to the average intensity across the two colours as measured by normalisation control probes present on the BeadChip. The methylation level at each CpG was expressed as a *β* value, which represents the fraction of methylated cytosines at that specific location.

Probes that were not detected in >20 % of the samples were excluded from the analyses. The analysis of other quality control measures provided by GenomeStudio suggested that the resulting filtered subset did not show any major quality issues. Missing data were first imputed using the *k*-nearest neighbours method as implemented in the R package “impute” for the principle components analysis only [36]. We then used the empirical Bayes method of Johnson et al. [37] (commonly referred to as “ComBat”) to minimise potential chip-specific batch effects. Lastly, in order to adjust the distributions of *β* values across probe type (Infinium I and II) and to enable joint analysis, we performed peak-based correction using two methods as described by Dedeurwaerder et al. [38] and Teschendorff et al. [39]. Because the peaks of type I and type II probes are well defined in our study samples, both methods performed sufficiently well. We opted for the beta-mixture quantile normalisation (BMIQ) method [39], for the main analyses.

### Probe and sample exclusions following quality control

Probe and sample exclusions are described in Additional file 3: Figure S2. In the EPIC cohort, the DNA methylation was measured at 485,577 loci on the genome in 166 cases and 166 matched controls before quality control exclusions. Sixty-five of these loci were SNPs, which were excluded from the analyses. Out of all 332 subjects, two subjects had to be excluded because of a diagnosis with another cancer prior to developing breast cancer and another two subjects because their matched pair was not located on the same chip. Following these initial sample exclusions, pre-processing of the DNA methylation data excluded 36,655 CpGs from the analyses because of missing values in >20 % of the samples and another three samples because of missing values for >5 % of the remaining CpGs. Finally, one sample (which formed an incomplete match pair) and 40,108 non-specific CpGs were excluded, resulting in 324 samples in which DNA methylation was measured at 408,749 CpGs. In the NOWAC cohort, DNA methylation was measured at 485,577 loci on the genome in all subjects: 192 cases and 192 matched controls. Sixty-five of these loci were SNPs, which were excluded from the analyses, as well as 224 CpGs after applying ComBat. We excluded 9 samples due to missing covariate data. Pre-processing of the DNA methylation data further excluded 28,459 CpGs from the analyses because of missing values in >20 % of the samples and another 14 samples because of missing values for >5 % of the remaining CpGs. Finally, 23 samples (which formed an incomplete match pair) and 40,417 non-specific CpGs were excluded, resulting in 338 samples (169 case-control pairs) in which DNA methylation was measured at 416,412 CpGs. Including only probes overlapping across the two datasets resulted in 407,455 probes.

### White blood cell type adjustment

Previous studies have highlighted the importance of taking the type of different WBCs into account when analysing DNA methylation in whole blood [40, 41]. WBC differentials were not available for our samples. To address this, we used HM450 methylation data obtained from purified CD4 T-cells, CD8 T-cells, CD19 B-cells, monocytes, natural killer (NK) cells, neutrophils and eosinophils, and whole PBMCs (*n* = 6 subjects) [41]. We identified the probes that differed significantly between each individual cell type and PBMC (linear regression using *β* values, *p* < 1e−07 and delta-*β* > 0.05). This identified *n* = 10,082 unique probes, which were subsequently removed from the statistical analyses, assuming as a first approach that blood composition only marginally affected methylation patterns at other sites (*n* = 444,054 remaining probes). Genome-wide estimation of cell composition was also used to infer cell proportions using the reference-based method [42] which did not change the results, rather than the reference-free adjustment method [43]. Methylation array data from the EPIC cohort is available at GEO with accession GSE51057.

### DNA methylation measurement, data pre-processing, and quality control for whole-genome bisulphite sequencing

DNA samples from the BGS cohort case-control study were stratified into four pathology sub-groups (in situ cases, ER-negative cases, ER-positive early onset <50 years, and ER-positive late onset >50 years, see Additional file 1: Table S4). Due to the high cost of whole genome sequencing, we used a pooling approach where incident breast cancer cases (*n* = 548) were pooled into 4 pools of DNA, and the matched healthy controls (*n* = 548) were pooled into matched pools. We pooled 200 ng of DNA from each subject into the 8 DNA pools that were then processed for WGBS using a published protocol for library preparation [44]. Libraries were sequenced using PE100bp reads using the HiSeq2500 with 2 lanes per library. Sequencing was conducted by the Institute of Cancer Research Tumour Profiling Unit. Data processing followed a standard pipeline: The quality of reads was analysed using SolexaQA [45]. Mate pairs were trimmed to 80 bp, reflecting a balance between uniquely mappable, high-quality reads. Bismark [46] was used to map trimmed read pairs to a bisulphite-converted representation of the hg19 (GRCh37) genome, using Bowtie 2. Bismark then calculated the proportion of methylated reads at each CpG site, after removing duplicated reads. This provided single nucleotide level resolution with approximately 50-fold coverage of ~14 million mappable CpG sites (13,903,531 CpGs). All subsequent analysis was performed in R, using “GRanges” package to generate coverage-weighted summary methylation values for different genomic categories/regions. We observed that the raw average methylation across CpG sites was dependent on coverage and therefore calculated a coverage-weighted mean methylation for each CpG site. Coverage-weighted mean was calculated with the following formula: Wmean = (M1*W1 + M2*W2 + M3*W3…)/sum(weights), where the CpG site was weighted (*w* = 1) if the coverage was greater than the median coverage in that pool and scaled down (*w* = 0.9, 0.8, 0.7, etc.) with each 10 % decrease in coverage from the median. We selected the 450k array CpG locations from the array annotation file and calculated coverage-weighted averages across all CpG sites that mapped to each genomic range and averaged across the CpG sites. We present the data from the CpG sites overlapping the 450k array for validation, with analysis of the whole data set to be reported elsewhere (Flanagan and Garcia-Closas, in preparation). We observed strong correlation between methylation values as measured by WGBS and Illumina 450k arrays for all probes (*R*^2^ > 0.97) and for probes with methylation values between 20 and 80 % methylated (*R*^2^ > 0.77).

### Statistical analysis

For the 450k array data, the mean *β* value across all probes was calculated for each sample as a measurement for epigenome-wide methylation, and a paired Wilcoxon test was used to assess differences between cases and controls. An age-adjusted estimate of the odds ratio of breast cancer was obtained from a conditional logistic regression model with case-control status as the outcome and the epigenome-wide methylation measurement as continuous predictor. We adjusted for age due to residual age differences between the controls that were matched to within 5 years in EPIC. The epigenome-wide methylation levels were categorised into quartiles based on the distributions in controls. As a quantitative measure of the overall methylation, each quartile was allocated its median value (pseudo-continuous variable). To ease comparison with the corresponding methylation distribution in controls, medians were centred and standardised using the observed mean and standard deviation over all probes investigated. Odds ratios for epigenome-wide methylation were estimated overall and by time between blood collection and diagnosis. Robust logistic regression was also used to confirm these results. We have performed the receiver operating curve (ROC) analysis to assess the classification performance of average DNA methylation levels to predict breast cancer case status. We report the odds ratios (ORs), 95 % confidence intervals (95 % CIs), and corresponding *p* values. *p* values <0.05 were considered to be statistically significant. B-spline logistic regression models fitted in the “bs” R package were used to explore the relationship between continuous measures of methylation levels and breast cancer risk and to estimate individual risk distribution. Meta-analysis was conducted using the “rmeta” R package and a random effects model for the summary estimate.

Probes were classified into different categories either reflecting their physical location in relation to CpG islands (island, shore, shelf) or based on a functional criterion (promoter, gene body, UTR, intergenic) according to the Illumina manifest file. CpG islands were classified as previously defined [47]. A CpG shore is defined as the area 2 kb on either side of the CpG island, and a CpG shelf is defined as the area 2 kb outside of the CpG shore [48, 12]. As in the work of Sandoval et al., we combined TSS200, TSS1500, 5′UTR, and 1st exon into a single “promoter” region [13]. Mean methylation over all probes within each category was calculated and ORs estimated, as described above.

Probe-wise analysis of 450k arrays was performed by first adjusting for technical confounding effects; DNA methylation levels at each CpG locus were adjusted using a generalised linear model (GLM) with beta-distributed response [49] including microarray and position on the microarray as technical confounders. Subsequently, to assess the association with case-control status, residuals from these models were entered as independent variable in a Poisson GLM with person-years of follow-up time as offset term and additionally adjusted for age at blood draw; this parameterisation yields results that are practically equivalent to those obtained using Cox proportional hazards model [50]. Multiple comparisons were taken into account by considering a Bonferroni-corrected significance threshold *α* = 0.05/407,455 ≈ 1.2 × 10^−7^.

## Additional files

Additional file 1:
**Supplementary Tables 1 to 11.** Tables include supporting information for analyses on EPIC and NOWAC cohorts, significant individual probes in EPIC and subject characteristics in EPIC, NOWAC and BGS cohorts used in this study. Additional file 2: Supplementary Figure 1. Methylation difference within case/control pairs by time to diagnosis in EPIC. Additional file 3: Supplementary Figure 2. Probe and Sample Filtering Steps in EPIC and NOWAC.
