# Supplementary material for: Epigenome-wide association study reveals decreased average methylation levels years before breast cancer diagnosis
Source: Clin Epigenetics. 2015 Aug 4;7(1):67. doi: 10.1186/s13148-015-0104-2 (PMC4524428; doi:10.1186/s13148-015-0104-2)
Supplement: Additional file 1: — Supplementary Tables 1 to 11. Tables include supporting information for analyses on EPIC and NOWAC cohorts, significant individual probes in EPIC and subject characteristics in EPIC, NOWAC and BGS cohorts used in this study. [file 13148_2015_104_MOESM1_ESM.doc]

**Epigenome-Wide Association Study reveals decreased average methylation levels years before breast cancer diagnosis**

Karin van Veldhoven ^1,2,&^, Silvia Polidoro ^2,&^, Laura Baglietto ^2^, Gianluca Severi ^2^, Carlotta Sacerdote ^2^, Salvatore Panico ^3^, Amalia Mattiello ^3^, Domenico Palli ^4^, Giovanna Masala ^4^, Vittorio Krogh ^5^, Claudia Agnoli ^5^, Rosario Tumino ^6^, Graziella Frasca ^6^, Kirsty Flower ^7^, Ed Curry ^7^, Nicholas Orr ^9^, Katarzyna Tomczyk ^9^, Michael E. Jones ^10^, Alan Ashworth ^8^, Anthony Swerdlow ^9,10^, Marc Chadeau-Hyam ^1^, Eiliv Lund ^11^, Montserrat Garcia-Closas^8,10^, Torkjel M. Sandanger ^11^, James M. Flanagan ^7#^, and Paolo Vineis ^1,2#^

**Supplementary Tables**

**Supplementary Table 1.** Blood cell Composition Characteristics in EPIC and NOWAC

**Supplementary Table 2.** Association between global methylation and breast cancer risk factors in EPIC

**Supplementary Table 3.** Association between average methylation and breast cancer risk in EPIC and NOWAC using only the overlapping probes in each data set (n=407,455).

**Supplementary Table 4.** Association between global methylation and breast cancer risk by CpG genomic feature per 1 SD in NOWAC

**Supplementary Table 5.** Association between global methylation and breast cancer risk factors in NOWAC

**Supplementary Table 6.** Association between principal components and subject variables in NOWAC

**Supplementary Table 7.** 26 strongest probe associations from conditional logistic regression analysis between DNA methylation and breast cancer in EPIC, sorted on effect size

**Supplementary Table 8.** Comparison of Subject Characteristics EPIC vs NOWAC

**Supplementary Table 9.** Subject Characteristics in EPIC

**Supplementary Table 10.** Subject Characteristics in NOWAC

**Supplementary Table 11.** Subject Characteristics in BGS

**Supplementary Table 1:** Blood cell Composition Characteristics in EPIC and NOWAC

|  | **Median Proportion % Cases** | **Median Proportion % Controls** | **Logistic Regression P-value (CaCo status)*** | **Coef **** | **95% low** | **95% high** | **p-value** |
| --- | --- | --- | --- | --- | --- | --- | --- |
| **EPIC** |  |  |  |  |  |  |  |
| **unadjusted** | **1.00** | **1.00** |  | **0.61** | **0.47** | **0.80** | **0.00039** |
| **Ref-adjusted** | **1.00** | **1.00** |  | **0.53** | **0.38** | **0.73** | **0.000151** |
| -Monocytes | 0.07 | 0.07 | 0.99 | 0.51 | 0.35 | 0.74 | 0.000396 |
| -B | 0.04 | 0.04 | 0.51 | 0.52 | 0.36 | 0.74 | 0.000398 |
| -CD4T | 0.11 | 0.13 | 0.22 | 0.50 | 0.35 | 0.71 | 0.000122 |
| -NK | 0.06 | 0.05 | 0.20 | 0.52 | 0.36 | 0.75 | 0.000399 |
| -CD8T | 0.13 | 0.14 | 0.74 | 0.51 | 0.36 | 0.72 | 0.00014 |
| -Granulocytes^ | 0.61 | 0.61 | 0.87 | 0.50 | 0.35 | 0.72 | 0.00022 |
|  |  |  |  |  |  |  |  |
| **NOWAC** |  |  |  |  |  |  |  |
| **unadjusted** | **1.00** | **1.00** |  | **1.03** | **0.82** | **1.30** | **0.81** |
| **Ref-adjusted** | **1.00** | **1.00** |  | **1.09** | **0.84** | **1.42** | **0.50** |
| -Monocytes | 0.08 | 0.08 | 0.89 | 1.10 | 0.84 | 1.45 | 0.49 |
| -B | 0.04 | 0.05 | 0.18 | 1.06 | 0.79 | 1.42 | 0.72 |
| -CD4T | 0.14 | 0.16 | 0.25 | 1.11 | 0.85 | 1.43 | 0.45 |
| -NK | 0.09 | 0.08 | 0.50 | 1.15 | 0.87 | 1.52 | 0.33 |
| -CD8T | 0.10 | 0.10 | 0.47 | 1.12 | 0.86 | 1.45 | 0.41 |
| Granulocytes^ | 0.58 | 0.58 | 0.71 | 1.12 | 0.85 | 1.47 | 0.42 |

* Logistic regression for case control status using the blood cell fraction proportion as the explanatory variable adjusting for age.

** Logistic regression for case control status and mean methylation adjusting for age and all blood cell fractions using N-1 approach for each blood cell fraction.

^ Granulocytes is the sum of Eosinophils and Neutrophils

**Supplementary Table 2.** Association between global methylation and breast cancer risk factors in EPIC

|  |  | **All probes** | **Probes on CpG islands** | **Probes on shores** | **Probes on shelves** | **Probes at open sea** |
| --- | --- | --- | --- | --- | --- | --- |
|  |  | **p-value** | **p-value** | **p-value** | **p-value** | **p-value** |
| **Age** | years | 0.83 | **0.001** | 0.95 | **0.03** | **0.05** |
| **Height** | cm | 0.59 | 0.47 | 0.48 | 0.99 | 0.98 |
| **Weight** | kg | 0.16 | 0.19 | 0.14 | 0.63 | 0.44 |
| **BMI (continuous)** | kg/m^2^ | 0.24 | 0.34 | 0.25 | 0.62 | 0.42 |
| **BMI (categorical)** | underweight | 0.56 | 0.57 | 0.66 | 0.35 | 0.74 |
|  | overweight | 0.81 | 0.76 | 0.90 | 0.71 | 0.53 |
|  | obese | 0.25 | 0.66 | 0.37 | 0.63 | 0.19 |
| **Physical activity (cat)** | moderately inactive | 0.32 | 0.16 | 0.27 | 0.91 | 0.95 |
|  | moderately active | 0.08 | 0.02 | 0.09 | 0.55 | 0.69 |
|  | active | 0.09 | 0.11 | 0.06 | 0.41 | 0.46 |
| **Alcohol** | gr/d | 0.89 | 0.26 | 0.78 | 0.60 | 0.41 |
| **Folate** | ug/d | **0.04** | 0.33 | **0.03** | 0.08 | 0.08 |
| **Smoking** | former | 0.08 | 0.09 | **0.04** | 0.48 | 0.55 |
|  | current | 0.35 | 0.09 | 0.16 | 0.75 | 0.66 |
| **Age at menarche (cat)** | 12-14 | 0.53 | 0.68 | 0.60 | 0.36 | 0.24 |
|  | ≥15 | 0.49 | 0.13 | 0.39 | 0.61 | 0.73 |
| **Age at menopause** | years | **0.02** | 0.23 | 0.06 | **0.03** | **0.05** |
| **Menopausal state** | pre/post- | 0.45 | **0.01** | 0.50 | 0.33 | 0.38 |
| **Ever pill** | yes/no | 0.21 | 0.77 | 0.24 | 0.06 | 0.08 |
| **Ever hrt** | yes/no | 0.47 | 0.47 | 0.89 | 0.12 | 0.08 |
| **ER status (cases only)** | positive/negative | 0.69 | 0.67 | 0.60 | 0.88 | 0.93 |
| **PR status (cases only)** | positive/negative | **0.01** | 0.20 | **0.009** | 0.15 | 0.10 |

**Supplementary Table 3.** Association between average methylation and breast cancer risk in EPIC and NOWAC using only the overlapping probes in each data set (n=407,455).

|  |  | **Cases**  **(n)** | **Controls (n)** | **OR** | **(95% CI)** | ***p*-value** |
| --- | --- | --- | --- | --- | --- | --- |
| **EPIC** |  |  |  |  |  |  |
| **By quartile** | *Q1 [0.529 – 0.546)* | 75 | 41 | 1.00 |  |  |
|  | *Q2 [0.546 – 0.549)* | 31 | 40 | 0.46 | (0.25 – 0.84) | 0.01 |
|  | *Q3 [0.549 – 0.551)* | 30 | 40 | 0.40 | (0.21 – 0.76) | 0.005 |
|  | *Q4 [0.551 – 0.560)* | 26 | 41 | 0.34 | (0.18 – 0.66) | 0.001 |
|  |  |  |  |  |  |  |
|  | *Per 1SD* | 162 | 162 | 0.61 | (0.46 - 0.80) | 0.0003 |
|  |  |  |  |  |  |  |
| **Time to Diagnosis** | *<3.8* | 81 | 81 | 0.66 | (0.46 – 0.94) | 0.02 |
| **(years)** | *>3.8* | 81 | 81 | 0.54 | (0.35 – 0.83) | 0.005 |
|  |  |  |  | *p het* = 0.48 |  |  |
|  |  |  |  |  |  |  |
| **NOWAC** |  |  |  |  |  |  |
| **By quartile** | *Q1 [0.527 – 0.538]* | 44 | 42 | 1.00 |  |  |
|  | *Q2 [0.538 – 0.540]* | 40 | 42 | 0.93 | (0.53 – 1.65) | 0.80 |
|  | *Q3 [0.540 – 0.543]* | 37 | 42 | 0.85 | (0.47 – 1.52) | 0.58 |
|  | *Q4 [0.543 – 0.551]* | 47 | 42 | 1.07 | (0.61 – 1.87) | 0.82 |
|  |  |  |  |  |  |  |
|  | Per 1SD | 168 | 168 | 1.01 | (0.81 – 1.28) | 0.90 |
|  |  |  |  |  |  |  |
| **Time to Diagnosis** | *<2.1* | 84 | 84 | 0.89 | (0.63 – 1.25) | 0.49 |
| **(years)** | *>2.1* | 84 | 84 | 1.14 | (0.83 – 1.58) | 0.41 |
|  |  |  |  | *p het* = 0.65 |  |  |
|  |  |  |  |  |  |  |

**Supplementary Table 4.** Association between global methylation and breast cancer risk by CpG genomic feature per 1 SD in NOWAC

|  |  | **# CpG loci** | **OR** | **(95% CI)** | ***p*-value** |
| --- | --- | --- | --- | --- | --- |
|  |  |  |  |  |  |
| **All** | Including all probes | 416,412 | 1.03 | (0.81 – 1.30) | 0.81 |
|  | *Excluding SNP probes* | 366,957 | 1.03 | (0.82 – 1.31) | 0.79 |
|  |  |  |  |  |  |
| **CpG Island** | *Island* | 127,193 | 1.11 | (0.87 – 1.43) | 0.40 |
|  | *Shores* | 100,256 | 1.11 | (0.86 – 1.42) | 0.42 |
|  | *Shelves* | 39,618 | 0.97 | (0.76 – 1.23) | 0.80 |
|  | *None* | 149,345 | 0.97 | (0.77 – 1.24) | 0.84 |
|  |  |  |  |  |  |
| **Gene Region Feature category** | *TSS1500* | 60,542 | 1.17 | (0.92 – 1.49) | 0.19 |
|  | *TSS200* | 44,518 | 1.03 | (0.79 – 1.34) | 0.84 |
|  | *5’UTR* | 37,547 | 1.09 | (0.86 – 1.40) | 0.47 |
|  | *1^st^ Exon* | 19,304 | 1.10 | (0.85 – 1.42) | 0.47 |
|  |  |  |  |  |  |
|  | *Promoter* | 83,995 | 1.01 | (0.78 – 1.32) | 0.92 |
|  | *Gene Body* | 141,077 | 1.01 | (0.79 – 1.28) | 0.94 |
|  | *UTR3* | 15,468 | 0.93 | (0.74 – 1.17) | 0.54 |
|  | *Intergenic* | 97,956 | 1.01 | (0.80 – 1.28) | 0.94 |
|  |  |  |  |  |  |

**Supplementary Table 5.** Association between global methylation and breast cancer risk factors in NOWAC

|  |  | **All probes** | **Probes on CpG islands** | **Probes on shores** | **Probes on shelves** | **Probes at open sea** |
| --- | --- | --- | --- | --- | --- | --- |
|  |  | **p-value** | **p-value** | **p-value** | **p-value** | **p-value** |
| **Age** | years | 0.85 | 0.87 | 0.55 | 0.69 | 0.75 |
| **Weight** | kg | 0.47 | 0.27 | 0.34 | 0.83 | 0.78 |
| **Height** | cm | 0.78 | 0.20 | 0.69 | 0.62 | 0.68 |
| **BMI (continuous)** | kg/m^2^ | 0.46 | 0.10 | 0.29 | 0.59 | 0.57 |
| **BMI (categorical)** | underweight | 0.35 | 0.76 | 0.67 | 0.46 | 0.42 |
|  | overweight | 0.07 | 0.42 | 0.09 | 0.16 | 0.17 |
|  | obese | 0.62 | 0.48 | 0.50 | 0.93 | 0.90 |
| **Smoking** | former | 0.64 | 0.97 | 0.80 | 0.71 | 0.75 |
|  | current | 0.14 | 0.22 | 0.03 | 0.50 | 0.65 |
| **Menopausal state** | pre/post-menopausal | 0.74 | 0.06 | 0.53 | 0.52 | 0.55 |
| **Ever hrt** | yes/no | 0.55 | 0.27 | 0.47 | 0.79 | 0.81 |
| **ER status (cases only)** | positive/negative | 0.36 | 0.60 | 0.40 | 0.45 | 0.50 |
| **PR status (cases only)** | positive/negative | 0.89 | 0.84 | 0.80 | 0.83 | 0.82 |

**Supplementary Table 6.** Association between principal components and subject variables in NOWAC

|  | **First PC** | **Second PC** | **Third PC** |
| --- | --- | --- | --- |
| **% of variance explained** | 0.097 | 0.040 | 0.020 |
| **Minimum *p*-value chips** | 0.06 | 0.48 | 0.23 |
| **Covariates** |  |  |  |
| **Case/control status** | 0.94 | 0.70 | 0.17 |
| **Age** | 0.74 | 0.67 | 0.93 |
| **Weight** | 0.75 | 0.15 | 0.78 |
| **Height** | 0.28 | 0.46 | 0.13 |
| **BMI (continuous)** | 0.87 | 0.07 | 0.33 |
| **BMI (categorical)** |  |  |  |
| *Underweight* | 0.56 | 0.61 | 0.79 |
| *Overweight* | 0.33 | 0.60 | 0.78 |
| *Obese* | 0.53 | 0.28 | 0.26 |
| **Smoking** |  |  |  |
| *Former* | 0.26 | 0.49 | 0.33 |
| *Current* | 0.41 | 0.69 | 0.47 |
| **Menopausal state** | 0.08 | **0.02** | 0.94 |
| **Ever hrt** | 0.74 | 0.18 | 0.35 |
| **ER status (cases only)** | 0.52 | 0.52 | 0.52 |
| **PR status (cases only)** | 0.73 | 0.73 | 0.73 |

To demonstrate there was no batch effect for chip we report the smallest *p*-value for

the association between the PCs and all chips.

**Supplementary Table 7:** 26 strongest associations from conditional logistic regression analysis between DNA methylation and breast cancer

in EPIC, sorted on effect size

| **Target ID** | ***p*-value**  **crude** | ***p*-value FDR**  **adjusted*** | **Average β-value**  **cases** | **Average β-value**  **controls** | **Effect size** | **CHR** | **Gene** | **Functional region** | **Relation to CGI** |
| --- | --- | --- | --- | --- | --- | --- | --- | --- | --- |
| cg17824939 | 7.30 x10^-8^ | 0.001486 | 0.6572 | 0.6911 | -0.03809 | 12 | *C12orf50* | 5’UTR |  |
| cg12486486 | 9.04 x10^-8^ | 0.001486 | 0.7635 | 0.7979 | -0.03787 | 4 | *YTHDC1* | Body |  |
| cg05455393 | 8.22 x10^-8^ | 0.001124 | 0.6913 | 0.7226 | -0.03097 | X | *FHL1* | TSS1500 | N_Shore |
| cg00124920 | 9.45 x10^-8^ | 0.001486 | 0.7716 | 0.7993 | -0.03079 | 1 | *C1orf220* | Body | S_Shelf |
| cg18038361 | 1.25 x10^-8^ | 0.001124 | 0.5463 | 0.5748 | -0.03050 | 18 | *TTR* | TSS1500 |  |
| cg03509901 | 1.01 x10^-8^ | 0.001124 | 0.8334 | 0.8607 | -0.02767 | 17 | *NLK* | 1stExon | S_Shore |
| cg04798824 | 6.72 x10^-8^ | 0.001486 | 0.7707 | 0.7996 | -0.02761 | 12 | *ANKS1B* | Body |  |
| cg06531158 | 3.01 x10^-8^ | 0.001340 | 0.8044 | 0.8314 | -0.02570 | 13 | *MIR548F5* | Body |  |
| cg17424007 | 8.42 x10^-8^ | 0.001486 | 0.7862 | 0.8078 | -0.02469 | 1 | *MYOG* | TSS1500 |  |
| cg20235510 | 5.91 x10^-8^ | 0.001124 | 0.5060 | 0.5288 | -0.02459 | 6 | *ZNF311* | Body |  |
| cg13140465 | 5.82 x10^-8^ | 0.001486 | 0.7399 | 0.7594 | -0.02241 | 15 | *NIPA1* | Body | N_Shore |
| cg16659470 | 1.37 x10^-8^ | 0.001124 | 0.6329 | 0.6564 | -0.02208 | 8 | *RBM12B* | 3’UTR |  |
| cg05343548 | 2.31 x10^-8^ | 0.001340 | 0.7651 | 0.7847 | -0.02176 | 20 |  |  | N_Shore |
| cg05567435 | 3.07 x10^-8^ | 0.001340 | 0.6763 | 0.6986 | -0.02015 | 12 | *METTL7B* | TSS1500 |  |
| cg24504843 | 8.29 x10^-8^ | 0.001486 | 0.7798 | 0.7980 | -0.01934 | 4 | *CEP135* | 3’UTR |  |
| cg10133171 | 4.86 x10^-8^ | 0.001450 | 0.8040 | 0.8268 | -0.01871 | 4 |  |  | N_Shore |
| cg26772788 | 3.61 x10^-8^ | 0.001340 | 0.7984 | 0.8182 | -0.01773 | 18 |  |  | N_Shelf |
| cg03338924 | 7.57 x10^-8^ | 0.001486 | 0.8365 | 0.8510 | -0.01720 | 6 | *PHACTR1* | Body | Island |
| cg04343242 | 8.25 x10^-8^ | 0.001486 | 0.7560 | 0.7737 | -0.01611 | 4 | *BDH2* | 3’UTR | S_Shelf |
| cg20640749 | 7.84 x10^-8^ | 0.001486 | 0.7209 | 0.7374 | -0.01439 | 6 | *GLP1R* | 3’UTR |  |
| cg01341572 | 3.28 x10^-8^ | 0.001340 | 0.9040 | 0.9147 | -0.01384 | 12 | *HNF1A* | TSS200 | Island |
| cg04599941 | 9.40 x10^-8^ | 0.001486 | 0.9264 | 0.9390 | -0.01316 | 5 | *SEMA5A* | Body |  |
| cg00962707 | 8.57 x10^-8^ | 0.001486 | 0.8728 | 0.8841 | -0.01254 | 16 |  |  | S_Shore |
| cg23494338 | 4.11 x10^-8^ | 0.001401 | 0.9061 | 0.9180 | -0.01106 | 10 |  |  | N_Shore |
| cg01836096 | 2.99 x10^-8^ | 0.001340 | 0.8930 | 0.9009 | -0.00918 | 8 | *C8ORFK29* | TSS200 | S_Shore |
| cg04187814 | 4.97 x10^-8^ | 0.001450 | 0.8978 | 0.9050 | -0.00846 | 15 | *KIAA1199* | 5’UTR |  |

* *p*-value adjusted for multiple testing using FDR

**Supplementary Table 8:** Comparison of Subject Characteristics EPIC vs NOWAC

|  |  | **EPIC** | **NOWAC** | **p-value &** |
| --- | --- | --- | --- | --- |
| **Age at blood collection** | median (range), yrs | 54.3 (34.7 – 70.4) | 55.4 (47.2 – 63.3) | **2.50e-06** |
| **Time to diagnosis** | median (range), yrs | 3.8 (0.04 – 15.7) | 2.1 (0.02 – 5.0) | **2.19e-15** |
| **Follow Up Time** | median (range), yrs | 8.9 (0.04 – 15.7) | 4.8 (3.1-6.6) | **<2.2e-16** |
| **Weight** | mean (range), kg | 65.3 (42.8 – 106.0) | 71.0 (47.0 – 105.0) | **9.49e-11** |
| **Height** | mean (range), cm | 158.9 (139.5 – 177.5) | 166.6 (143.0 – 180.0) | **<2.2e-16** |
| **BMI (continuous)** | mean (range) | 25.9 (15.9 – 45.3) | 25.6 (18.0 – 39.1) | 0.33 |
| **BMI (categorical)*** | n (%) |  |  |  |
| *Underweight* |  | 4 | 2 | 0.47 |
| *Normal* |  | 142 | 158 |  |
| *Overweight* |  | 123 | 126 |  |
| *Obese* |  | 53 | 43 |  |
| **Smoking*** | n (%) |  |  |  |
| *Never* |  | 191 | 120 | **7.81e-09** |
| *Former* |  | 64 | 127 |  |
| *Current* |  | 67 | 74 |  |
| **Menopausal state*** | n (%) |  |  |  |
| *pre-menopausal* |  | 122 | 47 | **1.25e-10** |
| *post-menopausal* |  | 199 | 265 |  |
| **Ever hrt*** | n (%) |  |  |  |
| *No* |  | 270 | 256 | 0.03 |
| *yes* |  | 51 | 76 |  |
| **ER status*** | n (%) |  |  |  |
| *Negative* |  | 18 | 26 | 0.34 |
| *Positive* |  | 56 | 130 |  |
| **PR status*** | n (%) |  |  |  |
| *Negative* |  | 27 | 33 | 0.86 |
| *Positive* |  | 44 | 60 |  |

& T-test for continuous variables, chi-squared test for categorical variables. P<0.01 highlighted in bold

**Supplementary Table 9:** Subject Characteristics in EPIC

|  |  | **Cases (n=162)** | **Controls (n=162)** |
| --- | --- | --- | --- |
| **Age at blood collection** | median (range), years | 54.4 (35.0 – 70.1) | 54.2 (34.7 – 70.4) |
| **Time to diagnosis** | median (range), years | 3.8 (0.04 – 15.7) |  |
| **Weight** | mean (range), kg | 64.5 (44.3 – 106.0) | 66.1 (42.8 – 104.6) |
| **Height** | mean (range), cm | 158.6(141.5 – 177.5) | 159.1 (139.5 – 175.5) |
| **BMI (continuous)** | mean (range) | 26.1 (18.1 – 41.1) | 25.6 (15.9 – 45.3) |
| **BMI (categorical)*** | n (%) |  |  |
| *Underweight* |  | 2 (1.2) | 2 (1.2) |
| *Normal* |  | 65 (40.1) | 77 (47.5) |
| *Overweight* |  | 68 (42.0) | 55 (34.0) |
| *Obese* |  | 26 (16.1) | 27(16.7) |
| **Physical activity (categorical)*** | n (%) |  |  |
| *Inactive* |  | 24 (14.8) | 9 (5.6) |
| *moderately inactive* |  | 35 (21.6) | 39 (24.1) |
| *moderately active* |  | 87 (53.7) | 101 (62.3) |
| *Active* |  | 14 (8.7) | 13 (8.0) |
| **Alcohol consumption** | mean (range) gr/d | 0.91 (0.00 – 68.0) | 2.78 (0.00 – 85.9) |
| **Folic acid intake** | mean (range) ug/d | 268.2 (97.4 – 637.8) | 262.4 (50.8 – 599.0) |
| **Smoking*** | n (%) |  |  |
| *Never* |  | 106 (65.5) | 85 (52.5) |
| *Former* |  | 23 (14.2) | 41 (25.3) |
| *Current* |  | 31 (19.1) | 36 (22.2) |
| **Age at menarche (categorical)*** | n (%) |  |  |
| *<12* |  | 26 (16.1) | 35 (21.6) |
| *12-14* |  | 121 (74.7) | 107 (66.1) |
| *≥15* |  | 13 (8.0) | 20 (12.3) |
| **Age at menopause (continuous)** | mean (range) years | 50.2 (38.0 – 57.0) | 49.1 (39.0 – 59.0) |
| **Menopausal state*** | n (%) |  |  |
| *pre-menopausal* |  | 60 (37.0) | 62 (38.3) |
| *post-menopausal* |  | 99 (61.1) | 100 (61.7) |
| **Ever pill*** | n (%) |  |  |
| *No* |  | 101 (62.3) | 95 (58.6) |
| *yes* |  | 59 (36.4) | 67 (41.4) |
| **Ever hrt*** | n (%) |  |  |
| *No* |  | 137 (84.6) | 133 (82.1) |
| *yes* |  | 23 (14.2) | 28 (17.3) |
| **ER status*** | n (%) |  |  |
| *Negative* |  | 18 (11.1) |  |
| *Positive* |  | 56 (34.6) |  |
| **PR status*** | n (%) |  |  |
| *Negative* |  | 27 (16.7) |  |
| *Positive* |  | 44 (27.2) |  |

*Numbers and percentages of subjects do not always add up to total number of subjects due to missing values

**Supplementary Table 10:** Subject Characteristics in NOWAC

|  |  | **Cases (n=168)** | **Controls (n=168)** |
| --- | --- | --- | --- |
| **Age at blood collection** | median (range),years | 55.4 (47.2 – 63.3) | 55.4 (47.2 – 63.2) |
| **Time to diagnosis** | median (range), years | 2.1 (0.02 – 5.0) |  |
| **Weight** | mean (range), kg | 70.5 (51.0 – 105.0) | 71.5 (47.0 – 100.0) |
| **Height** | mean (range), cm | 166.9 (143.0 – 180.0) | 166.4 (150.0 – 180.0) |
| **BMI (continuous)** | mean (range) | 25.4 (18.0 – 39.1) | 25.8 (18.4 – 35.7) |
| **BMI (categorical)*** | n (%) |  |  |
| *Underweight* |  | 1 (0.6) | 1 (0.6) |
| *Normal* |  | 86 (51.2) | 72 (42.9) |
| *Overweight* |  | 58 (34.5) | 68 (40.5) |
| *Obese* |  | 19 (11.3) | 24 (14.3) |
| **Smoking*** | n (%) |  |  |
| *Never* |  | 58 (34.5) | 62 (36.9) |
| *Former* |  | 66 (39.3) | 61 (36.3) |
| *Current* |  | 37 (22.0) | 37 (22.0) |
| **Menopausal state*** | n (%) |  |  |
| *pre-menopausal* |  | 23 (13.7) | 24 (14.3) |
| *post-menopausal* |  | 132 (78.6) | 133 (79.2) |
| **Ever hrt*** | n (%) |  |  |
| *No* |  | 112 (66.7) | 144 (85.7) |
| *yes* |  | 53 (31.5) | 23 (13.7) |
| **ER status*** | n (%) |  |  |
| *Negative* |  | 26 (15.5) |  |
| *Positive* |  | 130 (77.4) |  |
| **PR status*** | n (%) |  |  |
| *Negative* |  | 33 (19.6) |  |
| *Positive* |  | 60 (35.7) |  |

*Numbers and percentages of subjects do not always add up to total number of subjects due to missing values

**Supplementary Table 11:** Subject Characteristics in BGS

|  |  | **Cases (n=548)** | **Controls (n=548)** |
| --- | --- | --- | --- |
| **Age at blood collection** | mean (range),years | 52 (24 - 82) | 52 (23 - 82) |
| **Time to diagnosis**  **Pathology stratified Pools** | mean (range), years | 2.7 (1 - 7.4)* | - |
| *DCIS* |  | 123 | 123 |
| *ER negative* |  | 66 | 66 |
| *ER positive Early Onset* |  | 176 | 176 |
| *ER positive Late Onset* |  | 183 | 183 |

*Excludes two cases diagnosed 22 days and one case diagnosed two years prior to blood collection, identified from record updates after the initial selection into the study.
