# Supplementary figures and images for: Epigenome-wide association study reveals decreased average methylation levels years before breast cancer diagnosis
Source: Clin Epigenetics. 2015 Aug 4;7(1):67. doi: 10.1186/s13148-015-0104-2 (PMC4524428; doi:10.1186/s13148-015-0104-2)

# Methylation difference within case/control pairs by time to diagnosis

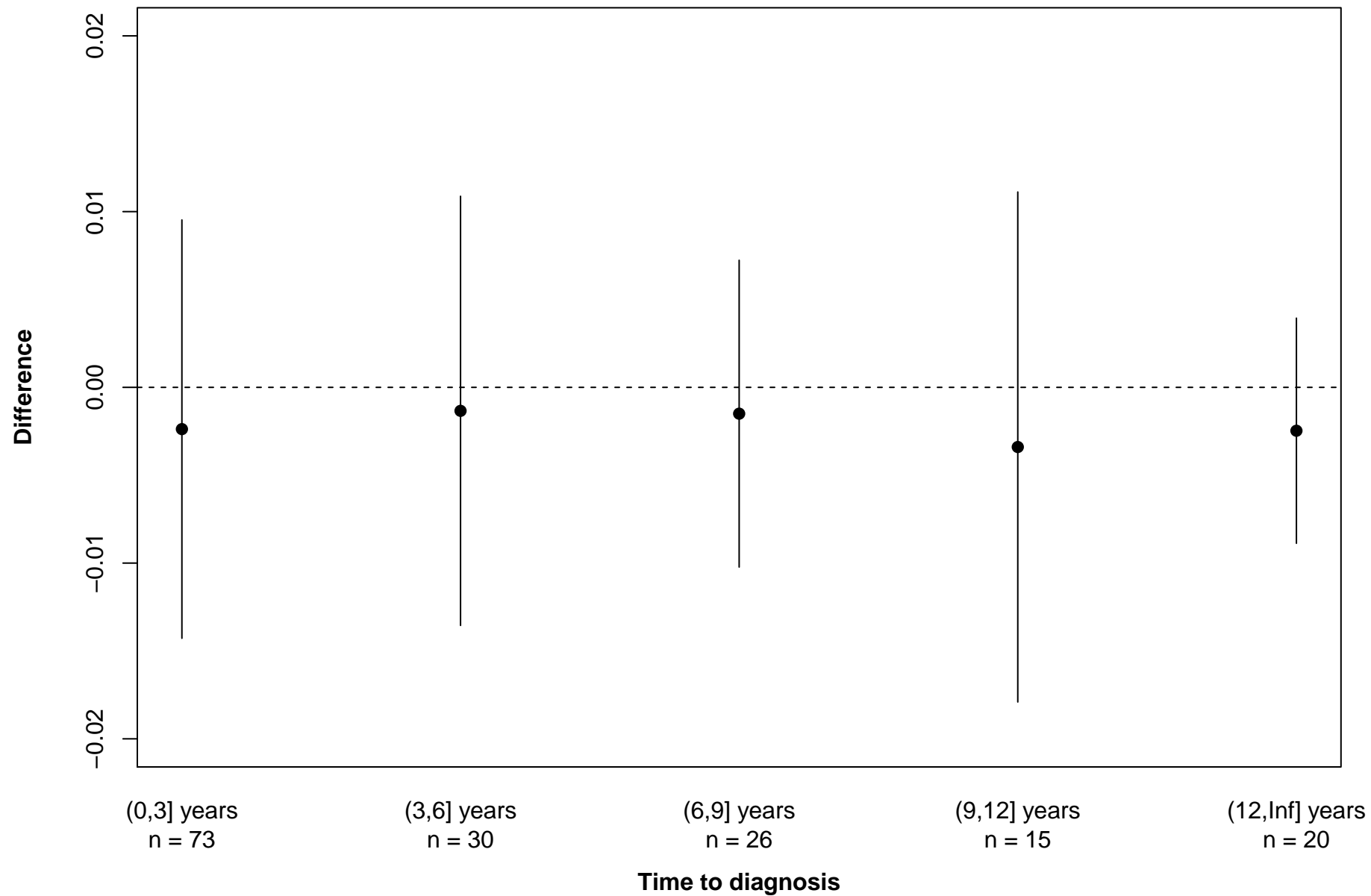

Supplement: Additional file 2: Supplementary Figure 1. — Methylation difference within case/control pairs by time to diagnosis in EPIC. [file 13148_2015_104_MOESM2_ESM.pdf]
