# Supplementary material for: Epigenome-wide association study reveals decreased average methylation levels years before breast cancer diagnosis
Source: Clin Epigenetics. 2015 Aug 4;7(1):67. doi: 10.1186/s13148-015-0104-2 (PMC4524428; doi:10.1186/s13148-015-0104-2)
Supplement: Additional file 3: Supplementary Figure 2. — Probe and Sample Filtering Steps in EPIC and NOWAC. [file 13148_2015_104_MOESM3_ESM.pdf]

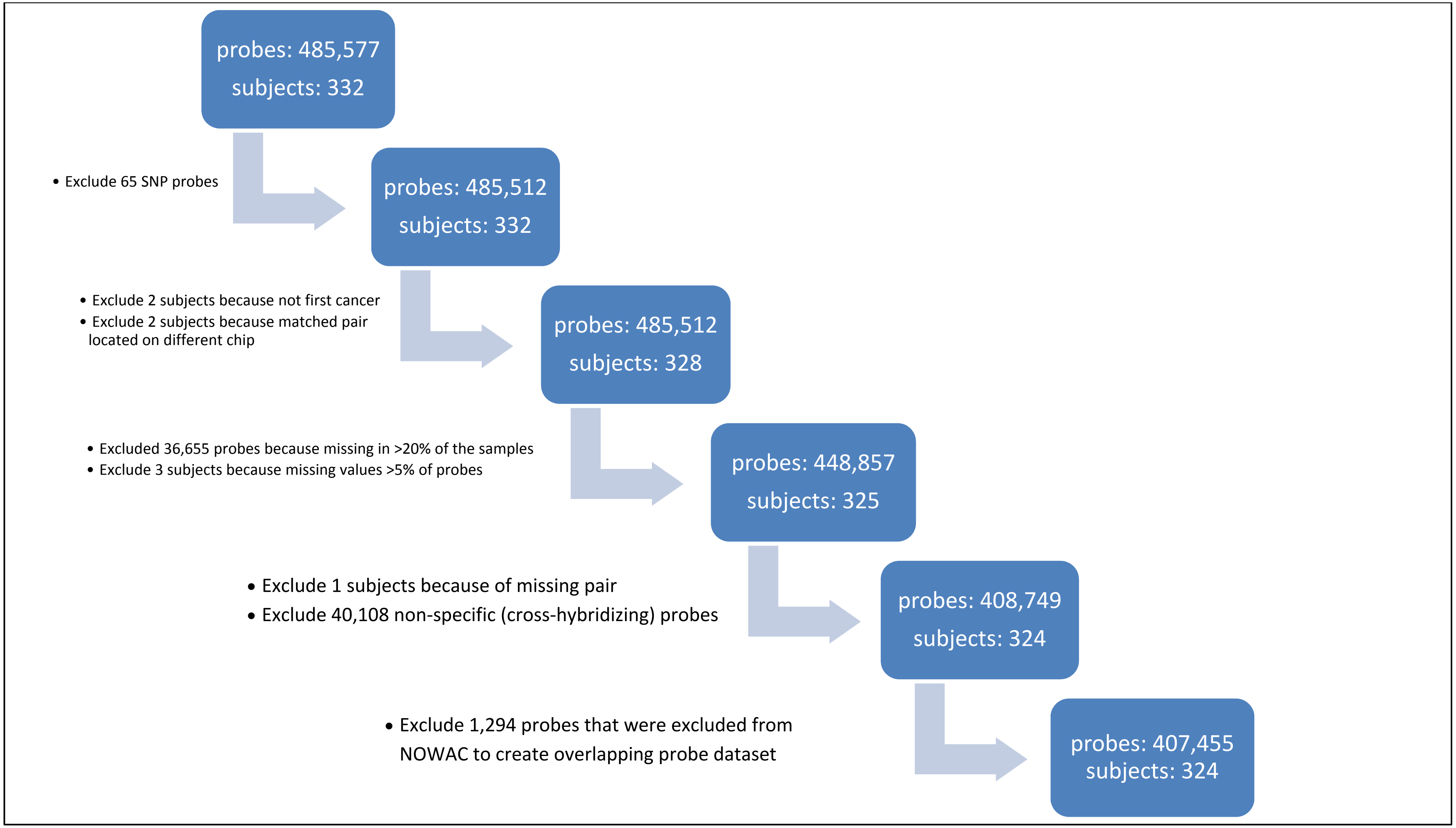

Supplementary Figure 1A: EPIC exclusion of probes and subjects

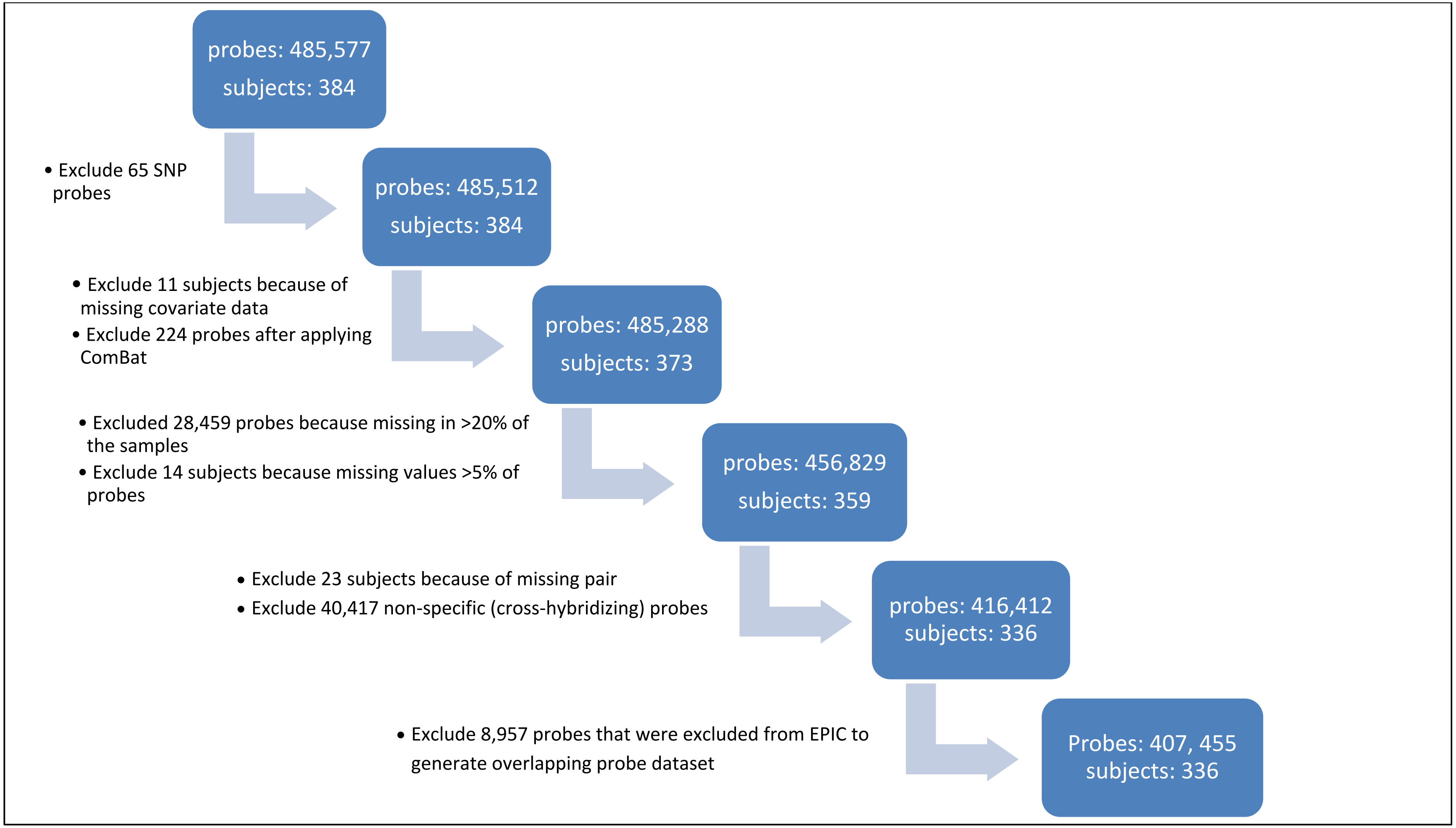

Supplementary Figure 1B: NOWAC exclusion of probes and subjects
